# Is the FVB/N mouse strain truly resistant to diet‐induced obesity?

**DOI:** 10.14814/phy2.13271

**Published:** 2017-05-08

**Authors:** Michelle Nascimento‐Sales, Izabelle Fredo‐da‐Costa, Adriane C. B. Borges Mendes, Suzane Melo, Thais T. Ravache, Thiago G. B. Gomez, Fernanda Gaisler‐Silva, Miriam O. Ribeiro, Arnaldo R. Santos, Marcela S. Carneiro‐Ramos, Marcelo A. Christoffolete

**Affiliations:** ^1^ Centro de Ciências Naturais e HumanasUniversidade Federal do ABCSanto AndréSão PauloBrazil; ^2^ Universidade Presbiteriana MackenzieSão PauloSão PauloBrazil

**Keywords:** C57Bl/6J, diet‐induced obesity, FVB/N, genetic background, glucose tolerance

## Abstract

C57Bl/6J mice are the gold standard animal model of diet‐induced obesity. These animals become obese with higher adiposity, blood fasting glucose, triglycerides, and total cholesterol when fed a high‐fat diet (HFD). Conversely, the FVB/N mouse line is thought to be resistant to diet‐induced obesity, with low or no weight gain and adiposity in response to a HFD. In this study, we investigated whether FVB/N mice are resistant or susceptible to metabolic disorder that is promoted by a HFD. Biometric parameters and blood chemistry were analyzed in C57Bl/6J and FVB/N mice that were fed a chow diet or HFD. Glucose and insulin sensitivity were assessed by performing the glucose tolerance test and measuring serum insulin/glucose and homeostasis model assessment‐insulin resistance. Metabolism‐related gene expression was investigated by real‐time reverse transcription polymerase chain reaction. Adipocyte morphology and liver steatosis were evaluated using standard histology. FVB/N mice had higher adiposity than C57Bl/6J mice that were fed a chow diet and were glucose intolerant. FVB/N mice that were fed a HFD presented higher insulin resistance and greater liver steatosis. Epididymal white adipose tissue exhibited severe inflammation in FVB/N mice that were fed a HFD. The FVB/N mouse strain is suitable for studies of diet‐induced obesity, and the apparent lack of a HFD‐induced response may reveal several strain‐specific events that are triggered by a HFD. Further studies of the FVB/N background may shed light on the complex multifactorial symptoms of obesity and metabolic syndrome.

## Introduction

Genetic background is an important variable in metabolic studies (Andrikopoulos et al. 2005). The C57Bl/6J mouse is the most widely used model in studies of obesity and considered highly susceptible to diet‐induced obesity (DIO), presenting increases in weight and fat body content when fed a high‐fat diet (HFD; (Buettner et al. 2007). This mouse line also develops insulin resistance (Shoelson et al. 2003), hypercholesterolemia, and higher serum triglycerides (TAGs) compared with their chow diet‐fed counterparts (for review, see (Buettner et al. 2007). FVB/N mice are a Swiss mouse‐derived line (Taketo et al. 1991). FVB/N mice are considered diet‐resistant because they do no present DIO when fed a HFD (Kim et al. 2013). The parameter that is measured to determine whether a particular strain is susceptible or resistant to DIO is white fat accumulation, specifically epididymal, omental, and surrounding kidney fat depots (Bjorndal et al. 2011). The lack of HFD‐induced obesity in FVB/N mice has discouraged investigators from using this diet‐resistant mouse line in studies of obesity and metabolic syndrome beyond tracking possible obesity‐prone‐related loci (Diament and Warden 2004).

Importantly, FVB/N mice are widely used to generate transgenic models because of the relative ease of manipulating oocytes and their higher number of offspring per litter (Taketo et al. 1991). This raises an issue concerning mouse backgrounds when using FVB/N transgenic mice in metabolic studies, which are largely performed using the C57BL/6J strain (Colombo et al. 2003; Doetschman 2009). Another important feature of the FVB/N mouse line is its well‐described glucose intolerance (Berglund et al. 2008; Haluzik et al. 2004).

In this study, we investigated the genetically distant FVB/N and C57BL/6J mouse lines (Beck et al. 2000) and their response to a HFD in a model of DIO. We analyzed biometric parameters, blood biochemistry, histological parameters, and gene expression. FVB/N were susceptible to the deleterious effects of a HFD, in which they developed higher glucose intolerance, pronounced hepatic steatosis, and severe inflammation in white adipose tissue (WAT).

## Material and Methods

### Animals

Male C57Bl/6J mice were obtained from CEDEME‐UNIFESP (Centro de Desenvolvimento de Modelos Experimentais para Medicina e Biologia – Universidade Federal de São Paulo). FVB/N mice were obtained from CEMIB‐Unicamp (Centro Multidisciplinar para Investigação Biológica na Área da Ciência de Animais de Laboratório – Universidade de Campinas). The animals were 4 weeks old and housed three per cage in ventilated cabinets (Alesco Ind. Com., Monte Mor, Campinas, SP, Brazil) under a 12 h/12 h light/dark cycle at 22°C ± 2°C with ad libitum access to water and a standard Nuvilab CR‐1 diet (Nuvital Nutrientes S/A, Colombo, PR, Brazil).

The animals were 8 weeks old at the beginning of the HFD. They were housed individually at 25°C ± 2°C and fed either chow (16.8% protein, 73.5% carbohydrate, and 4.8% fat; 4 kcal/g) or a HFD (23% protein, 35.5% carbohydrate, and 35.9% fat; 6 kcal/g; Rhoster Ind. Com. Ltda, Araçoiaba da Serra, SP, Brazil). Lard was used as the main source of fat. The experiment lasted 68 days, after which the animals were food‐deprived for 6 h and killed by cardiac puncture under urethane (1500 mg/kg) and *α*‐chloralose (100 mg/kg) anesthesia, which was twice the dose used by Dalkara et al. (1995). After blood collection, epididymal WAT (epiWAT), subWAT, brown adipose tissue (BAT), and the liver were harvested, weighed, and either snap‐frozen for the biomolecular studies or fixed in Baker's formalin (WAT) or TissueTek O.C.T. compound (liver; Sakura Finetek, Torrance, CA) for the histological analysis. The animal protocol was approved by CEUA‐UFABC (Comissão de Ética em Uso de Animais – Universidade Federal do ABC) in agreement with Brazilian Federal Law 11.794/2008. Two independent experiments were conducted, with a total of 12 animals per group. Because of technical difficulties (e.g., insufficient serum for all of the analyses) and equipment limitations (e.g., the maximum number of samples that could be analyzed simultaneously by real‐time quantitative polymerase chain reaction [RT‐qPCR]), the *n* is indicated for each parameter that was analyzed.

### Biometrics and blood chemistry

Animals and food were weighed every Monday, Wednesday, and Friday. Food consumption was calculated as the difference between the amount offered and leftover and is presented as cumulative food consumption per group. Serum total cholesterol and TAGs were determined by commercial kits (Labtest Diagnóstica S/A, Lagoa Santa, MG, Brazil). Insulin levels were determined by mouse insulin enzyme‐linked immunosorbent assay (ELISA; Mercodia AB, Uppsala, Sweden). Leptin levels were determined by mouse leptin ELISA (Elabscience Biotechnology, Bethesda, MD).

### Glucose tolerance test and serum insulin

On day 58 of the experiment, food was removed 4 h prior to the GTT. Blood was obtained from the tail tip. Glycemia was determined using the Accu‐Chek Active glucose monitor (Roche Diagnostics GmbH, Mannheim, Germany). After basal glycemia levels were determined at time 0, the animals received glucose (1 g/kg, i.p.), and glycemia was monitored at 30, 60, 90, and 120 min. Basal GTT time point serum insulin and basal blood glucose were used to calculate homeostasis model assessment‐insulin resistance (HOMA‐IR), and the quantitative insulin sensitivity check index (QUICKI); (Berglund et al. 2008; Cacho et al. 2008; Katz et al. 2000; Matthews et al. 1985) as a surrogate method to evaluate insulin sensitivity.

### Histological analysis

Paraffin‐embedded sections (7 *μ*m) of epiWAT were cut on an RM2235 microtome (Leica Biosystems Nussloch GmbH, Nubloch, Germany), stained with hematoxylin and eosin (H&E), and photographed at 200× magnification using an Axio Imager.A2m microscope (Carl Zeiss Microscopy GmbH, Jena, Germany). TissueTek O.C.T.‐embedded sections (7 *μ*m) of the liver were cut on a CM1860 cryostat (Leica), stained with H&E, and photographed at 100× magnification. For adipocyte area determination, images were loaded into Image‐Pro Plus 3.0 software (Media Cybernetics, Rockville, MD). Samples from five animals from each group were analyzed, and at least 100 cells were measured per animal. The minimal number of adipocytes that were measured per group was ~720 (chow‐fed FVB mice), and the maximal number was ~1150 (chow‐fed C57Bl/6J mice).

### Total RNA extraction and RT‐qPCR

Total RNA was extracted using Trizol reagent (Life Technologies, Carlsbad, CA). Total RNA (2.5 *μ*g) underwent cDNA synthesis using the High‐Capacity cDNA Reverse Transcription Kit (Life Technologies). cDNA was diluted 5×, and PCR was performed using the QuantiFast SYBR Green PCR kit (Qiagen GmbH, Düsseldorf, Germany) and a Rotor Gene‐Q device (Qiagen). The cycling conditions were 95°C for 5 min (hot start), 95°C for 15 sec, and 60°C for 1 min for 40 cycles. For mRNA expression analysis, the 2^−∆∆CT^ method was used (Livak and Schmittgen 2001). Primers are available upon request.

### Statistical analysis

The results are expressed as mean ± SEM. The data were analyzed using Prism 5 software (GraphPad, La Jolla, CA). Student's *t*‐test was used for comparisons between C57Bl/6J and FVB/N mice under chow diet conditions and for comparisons between the chow diet and HFD in C57Bl/6J and FVB/N mice. The area under the curve (AUC) was determined from the GTT plot. The distribution of the adipocyte area was plotted as relative frequencies at 500 *μ*m^2^ intervals.

## Results

### FVB/N mice are not resistant to weight gain but present lower epididymal fat accumulation

As expected, C57Bl/6J mice presented increases in body weight and weight gain in response to the HFD, although they had lower food and caloric intake. No difference was found in liver weight in response to the HFD in C57Bl5/J mice, but all fat depots were heavier (iBAT was ~1.6‐times larger, subWAT was ~3‐times larger, and epiWAT was ~3.4‐times larger; Table 1). Fasting serum leptin, insulin, glycemia, and cholesterol increased in response to the HFD in C57Bl/6J mice. Both HOMA‐IR and QUICK showed insulin resistance. Triglycerides did not rise in this model. Altogether, these data validated our DIO protocol.

**Table 1:** Biometrics and blood chemistry parameters in C57Bl/6J and FVB/N mice that were fed the chow diet or HFD

	C57BL6/J Chow			C57BL6/J HFD			FVB/N Chow			FVB/N HFD			C57BL6/J	FVB/N	C57BL6/J vs. FVB/N
	Mean	SEM	*n*	Mean	SEM	*n*	Mean	SEM	*n*	Mean	SEM	*n*	Chow vs. HFD	Chow vs. HFD	
Body weight (g)	27.0	0.69	12	38.9	0.89	12	32.4	1.35	12	43.3	0.86	12	*P* < 0.0001	*P* < 0.0001	*P* < 0.01
Body length (cm)	9.78	0.06	12	9.98	0.09	12	9.87	0.05	11	10.23	0.08	11	n.s.	*P* < 0.01	n.s.
Weight gain (g)	3.2	0.94	12	15.3	0.72	12	5.4	1.01	12	16.3	0.64	12	*P* < 0.0001	*P* < 0.0001	n.s.
Food intake (g)	280	3.5	12	188	3.8	12	328	4.3	12	237	9.4	12	*P* < 0.0001	*P* < 0.0001	*P* < 0.0001
Food intake (kcal)	1132	14	12	1050	21	12	1327	17	12	1321	52	12	*P* < 0.01	n.s.	*P* < 0.0001
Liver (g)	1.2	0.11	6	1.3	0.04	6	1.4	0.04	11	2.5	0.15	11	n.s.	*P* < 0.0001	n.s.
iBAT (g)	0.12	0.019	6	0.19	0.019	6	0.24	0.036	11	0.28	0.035	11	*P* < 0.05	n.s.	*P* < 0.05
subWAT (g)	0.75	0.10	6	2.2	0.21	6	2.4	0.02	4	3.5	0.09	4	*P* < 0.0001	*P* < 0.0001	*P* < 0.0001
epiWAT (g)	0.58	0.06	12	1.97	0.12	12	1.11	0.09	11	0.92	0.06	11	*P* < 0.0001	n.s.	*P* < 0.001
epiWAT/B.W (100^a^)	2.11	0.15	12	4.99	0.26	12	3.78	0.34	11	2.26	0.14	11	*P* < 0.0001	*P* < 0.001	*P* < 0.001
epiWAT/length (10^a^)	0.60	0.06	12	1.98	0.13	12	1.12	0.09	11	0.89	0.05	11	*P* < 0.0001	*P* < 0.05	*P* < 0.001
Leptin (pg/mL)	1575	367	11	5121	813	10	1521	238	6	3115	552	8	*P* < 0.01	*P* < 0.05	n.s.
Serum insulin (pmol/L)	245	47	11	682	127	11	355	113	11	1003	130	11	*P* < 0.01	*P* < 0.01	n.s.
Blood glucose (mmol/L)^a^	9.3	0.24	12	11.5	0.25	12	10.3	0.58	12	14.4	0.74	12	*P* < 0.0001	*P* < 0.001	n.s.
HOMA‐IR^a^	5.9	0.66	6	19	3.0	6	31	8.8	4	161	47	4	*P* < 0.01	*P* < 0.05	*P* < 0.01
QUICKI^a^	0.30	0.004	6	0.26	0.005	6	0.25	0.007	4	0.21	0.005	4	*P* < 0.001	*P* < 0.01	*P* < 0.001
Serum triglycerides (mg/dL)	27	1.7	6	35	7.9	5	97	10.5	7	55	4.3	8	n.s.	*P* < 0.01	*P* < 0.0001
Serum cholesterol (mg/dL)	129	4.2	6	163	7.2	5	166	7.2	7	185	6.5	8	*P* < 0.01	n.s.	*P* < 0.01

Basal glycemia, determined by GTT.

a Basal insulin and glycemia levels (determined by the GTT at time 0) were used to calculate HOMA‐IR and QUICKI.

The comparisons of C57Bl/6J and FVB/N mice that were fed the chow diet revealed comparable weight gain in FVB/N mice, despite their greater food and caloric intake (Table 1). No difference in body length or liver weight was found. All fat depots were heavier (~2‐times heavier for epiWAT, ~3‐times heavier for subWAT, and ~2‐times heavier for iBAT) in FVB/N mice compared with C57Bl/6J mice that were fed the chow diet (Table 1). Despite overall larger fat depots, serum leptin, insulin, and blood glucose were not different between strains. However, HOMA‐IR and QUICK indicated insulin resistance in FVB/N mice (Table 1). We also found higher fasting serum cholesterol and TAGs in FVB/N mice than in C57Bl/6J mice (Table 1).

FVB/N mice that were fed the HFD presented significant increases in weight gain and body weight compared with their chow‐fed counterparts. FVB/N mice that were fed the HFD were longer than their chow‐fed counterparts. Despite lower food intake, caloric intake was about the same. The liver, iBAT, and subWAT were ~1.8‐, ~1.16‐, and ~1.5‐times larger in FVB/N mice that were fed the HFD, whereas epiWAT was about the same size. Interestingly, the larger tissues did not account for the total difference in weight between FVB/N mice that were fed the chow diet and HFD. To better characterize weight gain, whole‐animal imaging, such as micro‐computed tomography, should be employed.

In FVB/N mice that were fed the HFD, fasting serum leptin, insulin, blood glucose, and cholesterol were higher than in their chow‐fed counterparts. Triglycerides were ~40% lower in response to the HFD (Table 1). In animals that were fed the HFD, HOMA‐IR and QUICK showed even higher insulin resistance compared with chow‐fed animals.

### FVB/N mice become more glucose intolerant when fed a high‐fat diet

**Figure 1:**
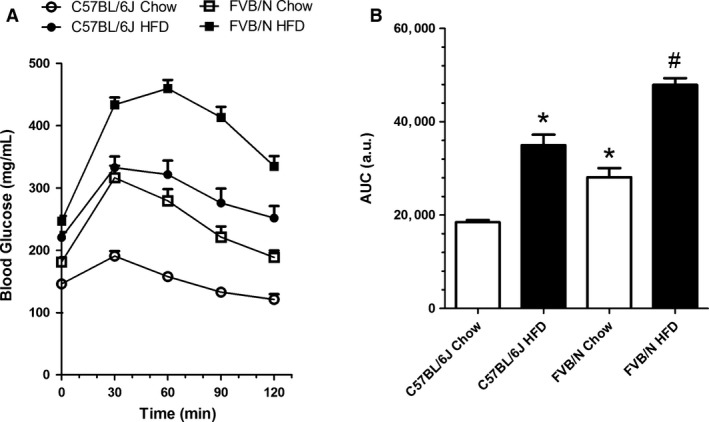
Glucose tolerance test (A) and AUC (B) in C57Bl/6J and FVB/N mice that were fed the chow diet or HFD. Glucose was injected at a dose of 1 g/kg (i.p.). *n* = 12, *P < 0.05, versus chow‐fed C57Bl/6J; ^#^ *P* < 0.05, versus chow‐fed FVB/N.

To further investigate glucose metabolism, we conducted the GTT. C57Bl/6J mice that were fed the HFD exhibited glucose intolerance (Fig. 1A), with a ~4‐times larger AUC (Fig. 1B) compared with chow‐fed animals. FVB/N mice exhibited glucose intolerance, with a ~3‐times larger AUC compared with C57Bl/6J mice (Fig. 1A and B), even under chow‐fed conditions.

Unexpectedly, FVB/N mice that were fed the HFD became even more glucose intolerant, with an AUC that was approximately 2‐times larger than their chow‐fed counterparts (Fig. 1A and B).

### FVB/N mice have larger adipocytes when fed a regular chow diet and severe inflammation when fed a HFD

**Figure 2:**
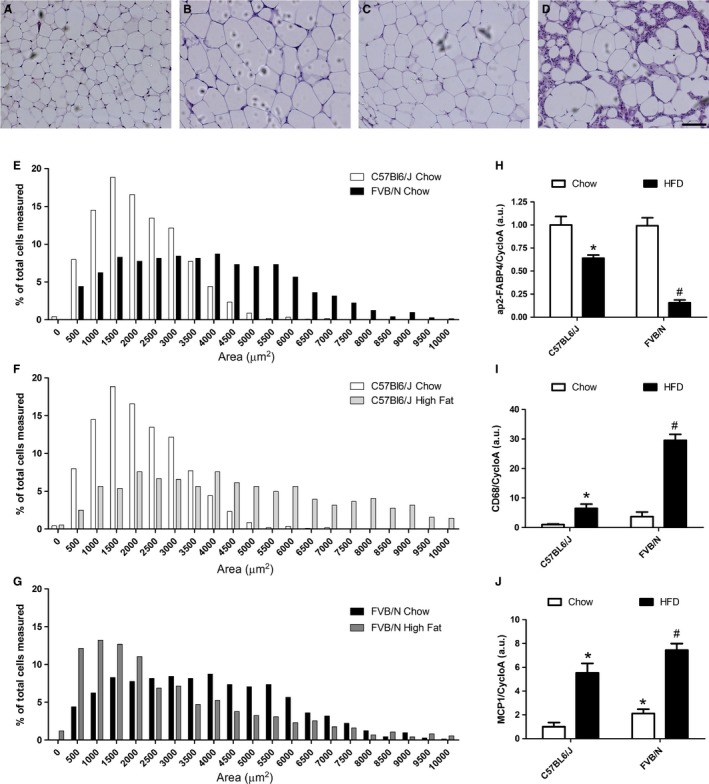
(A–D) Representative photomicrographs (200× magnification) of epiWAT in chow‐fed C57Bl/6J mice (A), HFD‐fed C57Bl/6J mice (B), chow‐fed FVB/N mice (C), and HFD‐fed FVB/N mice (D). (E–G) Relative frequencies of cross sectional area (CSA) in chow‐fed C57Bl/6J mice and chow‐fed FVB/N mice (E), chow‐ and HFD‐fed C57Bl/6J mice (F), and chow‐ and HFD‐fed FVB/N mice (G). (H–J) Gene expression of adipocyte differentiation marker (H) and inflammation (I, J) in chow‐fed C57Bl/6J mice, HFD‐fed C57Bl/6J mice, chow‐fed FVB/N mice, and HFD‐fed FVB/N mice. *P < 0.05, versus chow‐fed C57Bl/6J; ^#^ *P* < 0.05, versus chow‐fed FVB/N mice. Scale bar = 100 *μ*m. *Fabp4* (ap2/FABP4), adipocyte protein 2/fatty acid binding protein 4; *Cd68*, CD68 antigen; *Ccl2* (MCP1), macrophage chemoattractant protein‐1; *Ppia*, cyclophilin A. *n* = 5 for relative frequencies. *n* = 9 for RT‐qPCR.

To better understand the apparent lack of a response of the epiWAT depot to the HFD, we histologically analyzed epiWAT. We found an increase in the adipocyte cross sectional area (CSA) of epiWAT in C57Bl/6J mice that were fed the HFD (Fig. 2B) compared with chow‐fed animals (Fig. 2A), which is consistent with a previous study (Wu et al. 2016). The distribution of CSAs is shown in Fig. 2F. The size distribution in chow‐fed C57Bl/6J mice shifted to a broader range in HFD‐fed animals (Fig. 2F). Chow‐fed FVB/N mice also had larger adipocytes compared with chow‐fed C57Bl/6J mice (Fig. 2A and C). The distribution of CSAs is shown in Figure 2E, in which the clustered distribution in chow‐fed C57Bl/6J mice is contrasted by the broader range that was observed in chow‐fed FVB/N mice.

Additionally, FVB/N mice that were fed the HFD presented severe tissue disorganization and inflammation (Fig. 2D). The average size distribution also changed, and adipocytes became smaller, although larger adipocytes were still found in the tissue (Fig. 2G).

To confirm the histological findings, we investigated the expression of *Fabp4* (ap2/FABP4) and *Cd68* and *Ccl2* (MCP‐1), markers of mature adipocytes and macrophages. In C57Bl/6J mice, we observed a decrease in *Fabp4* (ap2/FABP4) mRNA levels in response to the HFD (Fig. 2H), accompanied by ~6.5‐ and 5.5‐times upregulation of *Cd68* and *Ccl2* (MCP‐1) mRNA levels (Fig. 2I and J). These results are consistent with the well‐established pattern of WAT inflammation in response to a HFD (Xu et al. 2003). Interestingly, a similar pattern was observed in FVB/N mice that were fed the chow diet, in which *Fabp4* (ap2/FABP4) mRNA levels were unaltered, and 2.1‐times upregulation of *Ccl2* (MCP‐1) mRNA levels was observed compared with chow‐fed C57Bl/6J mice. *Cd68* mRNA levels were not significantly different.

*Fabp4* (ap2/FABP4) mRNA levels were ~6.4‐times downregulated in epiWAT in FVB/N mice that were fed the HFD compared with their chow‐fed counterparts (Fig. 2H). *Cd68* mRNA levels increased nearly 8‐times in epiWAT in HFD‐fed FVB/N mice (Fig. 2I). *Ccl2* (MCP‐1) gene expression increased ~3.5‐times (Fig. 2J). These findings corroborated the severe inflammation that was seen in epiWAT in HFD‐fed FVB/N mice. These results indicate that the HFD had highly deleterious effects in FVB/N mice.

### epiWAT collapse in FVB/N mice is further supported by RT‐PCR analysis

To further investigate the severity of inflammation and molecular changes that were caused by the HFD in the two mouse lines, we analyzed key transcription factors, fatty acid and glucose metabolism‐related genes, and important adipokines. In C57Bl/6J mice, the HFD downregulated the transcription factors *Pparg2* (PPAR*γ*2), *Pgc1a* (PGC1*α*), and *Cebpa* (C/EBP*α*) ~1.7‐, 3.3‐, and 1.7‐times, respectively. *Srebp1* (SREBP‐1c) expression was unchanged (Table 2).

**Table 2:** Gene expression in epiWAT in C57Bl/6J and FVB/N mice that were fed the chow diet or HFD. *Pparg2* (PPAR*γ*2), peroxisome proliferator‐activated receptor *γ*2; *Pgc1a* (PGC1α), peroxisome proliferative activated receptor *γ*, coactivator 1*α*;* Cebpa* (C/EBP*α*), CCAAT/enhancer‐binding protein *α*;* Srebp1* (SREBP‐1c), sterol regulatory element‐binding transcription factor 1c; *Acaca* (ACC*α*, acetyl‐CoA carboxylase *α*;* Fasn* (FAS), fatty acid synthase; *Cpt1a* (CPT‐I), carnitine palmitoyltransferase I; *Lipe* (HSL), hormone‐sensitive lipase; *Ucp2*, uncoupling protein 2; *Pfk‐1*, phosphofructokinase 1; *Pck1* (PEPCKcit), phosphoenol pyruvate kinase cytosolic; *Slc2a4* (GLUT4), glucose transporter type 4; *Lep*, leptin; *Tnf* (TNF*α*, tumor necrosis factor *α*;* Il6* (IL6), interleukin 6. *n* = 9

	C57BL6/J Chow		C57BL6/J HFD		FVB/N Chow		FVB/N HFD		C57BL6/J	FVB/N	C57BL6/J vs. FVB/N
	Mean	SEM	Mean	SEM	Mean	SEM	Mean	SEM	Chow vs. HFD	Chow vs. HFD	
Transcriptional factors											
*Pparg2* (PPAR*γ*2)	1.0	0.05	0.6	0.09	1.1	0.11	0.2	0.02	*P* < 0.01	*P* < 0.0001	n.s.
*Pgc1a* (PGC1*α*)	1.0	0.14	0.3	0.05	0.8	0.13	0.3	0.08	*P* < 0.001	*P* < 0.01	n.s.
*Cebpa* (C/EBP*α*)	1.0	0.07	0.6	0.05	0.8	0.07	0.2	0.02	*P* < 0.0001	*P* < 0.0001	n.s.
*Srebp1* (SREBP‐1c)	1.0	0.11	0.9	0.09	1.4	0.13	2.1	0.23	n.s.	*P* < 0.05	*P* < 0.05
Fatty acid metabolism											
*Acaca* (ACC*α*)	1.0	0.20	0.3	0.06	2.3	0.50	0.2	0.04	*P* < 0.01	*P* < 0.001	*P* < 0.05
*Fasn* (FAS)	1.0	0.26	0.4	0.09	2.5	0.54	0.2	0.05	*P* < 0.05	*P* < 0.001	*P* < 0.05
*Cpt1a* (CPT‐I)	1.0	0.24	1.7	0.20	2.8	0.29	6.2	0.73	*P* < 0.05	*P* < 0.001	*P* < 0.001
*Lipe* (HSL)	1.0	0.08	0.6	0.08	1.0	0.13	0.1	0.02	*P* < 0.01	*P* < 0.0001	n.s.
*Ucp2*	1.0	0.14	3.0	0.49	3.7	0.45	9.2	0.88	*P* < 0.01	*P* < 0.0001	*P* < 0.0001
Glucose metabolism											
*Pfk‐1*	1.0	0.07	0.8	0.07	1.5	0.19	0.5	0.08	n.s.	*P* < 0.001	*P* < 0.05
*Pck1* (PEPCKcit)	1.0	0.14	0.3	0.06	1.1	0.18	0.02	0.01	*P* < 0.001	*P* < 0.0001	n.s.
*Slc2a4* (GLUT4)	1.0	0.06	0.4	0.06	1.2	0.22	0.05	0.01	*P* < 0.0001	*P* < 0.0001	n.s.
Adipokines											
*Lep* (Leptin)	1.0	0.16	3.4	0.52	2.3	0.37	0.7	0.18	*P* < 0.001	*P* < 0.01	*P* < 0.01
*Tnf* (TNF*α*)	1.0	0.38	4.8	1.74	1.9	0.39	19	2.84	*P* < 0.05	*P* < 0.0001	n.s.
*Il6* (IL6)	1.0	0.49	4.1	0.76	2.8	0.40	9.0	1.71	*P* < 0.01	*P* < 0.01	*P* < 0.05

With regard to fatty acid metabolism, the HFD downregulated *Acaca* (ACC*α*), *Fasn* (FAS), and *Lipe* (HSL) ~3.3‐, 2.5‐, and 1.7‐times, respectively. *Cpt1a* (CPT‐I) and *Ucp2* expression increased 1.7‐ and 3‐times, respectively, suggesting fatty acid oxidation in the tissue. With regard to glucose metabolism, the HFD decreased *Pck1* (PEPCKcit) and *Slc2a4* (GLUT4) expression 3.3‐ and 2.5‐times, respectively. As expected, the HFD upregulated *Lep* (leptin), *Tnf* (TNF*α*), and *Il6* (IL6) 3.4‐, 4.8‐, and 4.1‐times, respectively, in C57Bl/6J mice.

The comparisons of the two mouse strains that were fed the chow diet indicated that *Srebp1* (SREBP1c) was upregulated 1.4‐times in FVB/N mice, but the other transcription factors remained unchanged (Table 2). The mRNA levels of the de novo lipogenic genes *Acaca* (ACC*α*) and *Fasn* (FAS) increased 2.3‐ and 2.5‐times, respectively (Table 2). The mRNA levels of the fatty acid oxidation‐related genes *Cpt1a* (CPT‐I) and Ucp2 also increased (2.8‐ and 3.7‐times, respectively). *Lipe* (HSL) expression was the same in both mouse lines. Glycolysis also appeared to be stimulated in epiWAT in FVB/N mice, in which *Pfk‐1* expression increased 1.5‐times. *Pck1* (PEPCKcit) and *Slc2a4* (GLUT4) expression was unchanged (Table 2).

Consistent with the histological findings, larger adipocytes were observed in epiWAT in FVB/N mice, which correlated well with the increases in the mRNA levels of *Lep* (Leptin) and *Il6* (IL6; 2.3‐ and 2.8‐times increases, respectively). No changes in *Tnf* (TNF*α* expression were observed.

The strong suppression of WAT transcription factors was observed in FVB/N mice that were fed the HFD, in which *Pparg2* (PPAR*γ*2) decreased 5.5‐times, *Pgc1a* (PGC1*α*) decreased 2.7‐times, and *Cebpa* (C/EBP*α*) decreased 4‐times. *Srebp1* (SREBP1c) expression was upregulated 2.1‐times (Table 2). De novo lipogenesis also appeared to be suppressed. *Acaca* (ACC*α*), *Fasn* (FAS), and *Lipe* (HSL) mRNA levels decreased ~11.5‐, 12.5‐, and 10‐times, respectively, whereas *Cpt1a* (CPT‐I) and *Ucp2* mRNA levels increased ~2.2‐ and 2.5‐times, respectively (Table 2). These results suggest higher fatty acid oxidation within the tissue.

Glycolysis also appeared to be impaired. *Pfk‐1* and *Slc2a4* (GLUT4) expression decreased ~3‐ and 55‐times, respectively. *Pck1* (PEPCKcit) mRNA levels decreased 110‐times. *Lep* (Leptin) expression decreased ~3.3‐times. *Tnf* (TNF*α*) and *Il6* (IL6) expression increased ~10‐ and 3.2‐times, respectively. To determine whether such gene regulation is accompanied by changes in protein levels or enzymatic activity, further studies that evaluate isolated mature adipocytes are required.

### FVB/N mice develop pronounced liver steatosis when fed a high‐fat diet

**Figure 3:**
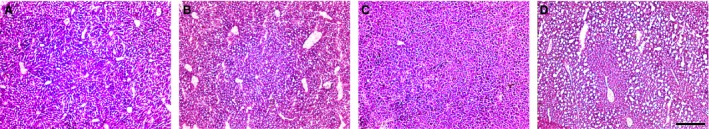
Representative photomicrographs (100× magnification) of the liver in chow‐fed C57Bl/6J mice (A), HFD‐fed C57Bl/6J mice (B), chow‐fed FVB/N mice (C), and HFD‐fed FVB/N mice (D). Scale bar = 250 *μ*m.

Adipocytes shrunk in epiWAT in FVB/N mice that were fed the HFD, and tissue was highly inflamed. We assumed that the higher fatty acid load would be taken up by the liver or other adipose depots. We processed the liver from five animals in each group for histological analysis and found this expected response of the liver of C57Bl/6J mice. Liver fat content increased in HFD‐fed C57Bl/6J mice (Fig. 3A and B), which is consistent with the literature (Fraulob et al. 2010). No histological differences were observed between chow‐fed FVB/N mice and chow‐fed C57Bl/6J mice (Fig. 3A and C). Unexpectedly, the liver in FVB/N mice that were fed the HFD presented pronounced lipid accumulation, indicating liver steatosis (Ueta et al. 2012); Fig. 3D).

### FVB/N mouse liver steatosis does not depend on de novo lipogenesis or re‐esterification gene regulation

To gain further insights into the histological findings, we investigated the expression of de novo lipogenesis genes (Strable and Ntambi 2010), TAG re‐esterification (Coleman and Lee 2004), fatty acid oxidation (Strable and Ntambi 2010), cholesterol metabolism, glucose metabolism, and lipid transport (Strable and Ntambi 2010).

Of the 24 genes analyzed, we found that 11 genes were responsive to the HFD in C57Bl/6J mice, which were all downregulated. *Srebp1* (SREBP1c), LXR*α*,* Acaca* (ACC*α*), *Scd1*,* Dgat‐2*,* Cpt1a* (CPT‐I), *Pgc1a* (PGC1*α*), *Insr* (insulin receptor), *Irs2* (IRS2), *Apob* (apoB), and *Mttp* were downregulated in HFD‐fed C57Bl/6J mice compared with their chow‐fed counterparts. *Acaca* (ACC*α*) expression is the first step in fatty acid synthesis (Strable and Ntambi 2010), and *Scd1* has been shown to play a major role in TAG biosynthesis (Strable and Ntambi 2010). Liver de novo lipogenesis appeared to be downregulated in HFD‐fed C57BL/6J mice (Table 3).

**Table 3:** Gene expression in the liver in C57Bl/6J and FVB/N mice that were fed the chow diet or HFD. *Nr1h3* (LXR*α*), liver x receptor *α*;* Scd1*, stearoyl CoA desaturase 1; *Dgat*‐*1*&*2*, diacylglycerol acyltransferase 1&2; *Ppara* (PPAR*α*, peroxisome proliferator‐activated receptor *α*;* Acox1*&*2*, acylCoA oxidase 1&2; *Hmgcr*, HMG‐CoA reductase; *Insig1*&*2*; insulin‐induced gene 1&2; *Insr* (insulin receptor); *Irs1*&*2*, insulin receptor substrate 1&2; *Apob* (apoB), apolipoprotein B; *Mttp*, microsomal triglyceride transfer protein. *n* = 9

	C57BL6/J Chow		C57BL6/J HFD		FVB/N Chow		FVB/N HFD		C57BL6/J	FVB/N	C57BL6/J vs. FVB/N
	Mean	SEM	Mean	SEM	Mean	SEM	Mean	SEM	Chow vs. HFD	Chow vs. HFD	
de novo Lipogenesis											
*Srebp1* (SREBP‐1c)	1.0	0.14	0.6	0.06	0.8	0.11	0.9	0.14	*P* < 0.05	n.s.	n.s.
*Nr1h3* (LXR*α*)	1.0	0.05	0.7	0.07	0.9	0.05	0.9	0.04	*P* < 0.01	n.s.	n.s.
*Acaca* (ACC*α*)	1.0	0.19	0.4	0.06	0.6	0.08	0.5	0.16	*P* < 0.05	n.s.	n.s.
*Fasn* (FAS)	1.0	0.37	0.3	0.04	0.4	0.05	0.4	0.25	n.s.	n.s.	n.s.
*Scd1*	1.0	0.28	0.2	0.06	0.6	0.09	0.05	0.03	*P* < 0.05	*P* < 0.0001	n.s.
Triglyceride re‐esterification											
*Dgat‐1*	1.0	0.07	0.8	0.06	1.1	0.12	1.3	0.15	n.s.	n.s.	n.s.
*Dgat‐2*	1.0	0.07	0.6	0.09	1.7	0.25	2.2	0.27	*P* < 0.01	n.s.	*P* < 0.05
Fatty acid oxidation											
*Cpt1a* (CPT‐I)	1.0	0.10	0.6	0.06	0.7	0.18	0.9	0.21	*P* < 0.01	n.s.	n.s.
*Ppara* (PPAR*α*)	1.0	0.13	1.0	0.13	1.4	0.11	1.7	0.14	n.s.	n.s.	*P* < 0.05
*Pgc1a* (PGC1*α*)	1.0	0.13	0.6	0.11	0.8	0.09	1.0	0.10	*P* < 0.05	n.s.	n.s.
*Acox1*	1.0	0.20	1.3	0.18	1.4	0.29	2.0	0.35	n.s.	n.s.	n.s.
*Acox2*	1.0	0.07	1.1	0.11	1.2	0.09	1.8	0.08	n.s.	*P* < 0.001	*P* < 0.05
Cholesterol metabolism											
*Hmgcr* (HMGCoA reductase)	1.0	0.24	1.4	0.24	0.5	0.11	0.9	0.24	n.s.	n.s.	n.s.
*Insig1*	1.0	0.27	0.9	0.18	1.0	0.11	1.3	0.17	n.s.	n.s.	n.s.
*Insig2*	1.0	0.21	1.0	0.20	1.4	0.21	2.6	0.29	n.s.	*P* < 0.01	n.s.
Glucose metabolism											
*Cebpa* (C/EBP*α*)	1.0	0.06	0.8	0.10	1.4	0.12	1.5	0.12	n.s.	n.s.	*P* < 0.05
*Pfk‐1*	1.0	0.13	1.1	0.11	1.6	0.27	2.2	0.35	n.s.	n.s.	n.s.
*Pck1* (PEPCKcit)	1.0	0.28	0.5	0.10	0.8	0.23	0.6	0.13	n.s.	n.s.	n.s.
*Insr* (insulin receptor)	1.0	0.10	0.6	0.08	0.7	0.06	0.8	0.06	*P* < 0.01	n.s.	*P* < 0.05
*Irs1*	1.0	0.14	0.7	0.06	1.2	0.12	1.4	0.13	n.s.	n.s.	n.s.
*Irs2*	1.0	0.13	0.4	0.07	0.9	0.12	0.6	0.08	*P* < 0.001	*P* < 0.05	n.s.
Other											
*Ucp2*	1.0	0.13	1.6	0.34	1.1	0.11	1.6	0.24	n.s.	n.s.	n.s.
*Apob* (apoB)	1.0	0.11	0.5	0.07	0.6	0.11	0.7	0.15	*P* < 0.01	n.s.	*P* < 0.05
*Mttp*	1.0	0.14	0.6	0.09	1.6	0.46	2.0	0.39	*P* < 0.05	n.s.	n.s.

Cholesterol metabolism appeared to be unchanged, in which the cholesterol biosynthesis rate‐limiting enzyme HMG‐CoA reductase (Tobert 2003) and the *Insig1* and *Insig2* genes were unaltered by the HFD (Table 3). These genes are well known to limit cholesterol biosynthesis (Goldstein et al. 2006).

Glucose metabolism also appeared to be preserved in C57BL/6J mice that were fed the HFD. In contrast, insulin signaling decreased, in which the insulin receptor and substrate, *Irs2*, were downregulated in C57BL/6J mice that were fed the HFD (Table 3).

The HFD also downregulated *Apob* (apoB) gene expression by nearly 50% compared with chow‐fed C57BL/6J mice (Table 3). apoB is involved in exporting lipids (e.g., phospholipids, triacylglycerol, and cholesteryl ester) from hepatocytes (Yao et al. 1997), and lower *Apob* expression may have led to lipid accumulation in the liver in HFD‐fed C57BL/6J mice. A similar pattern was observed for *Mttp*, the expression of which decreased ~1.7‐times. MTTP plays an important role in apoB secretion (Raabe et al. 1999).

*Ppara* (PPAR*α*), *Acox1*, and *Acox2* were unaltered by the HFD, suggesting no changes in fatty acid oxidation in the liver. *Pgcc1a* (PGC1*α*) is another important component of fatty acid oxidation, which was downregulated ~1.7‐times (Table 3). A similar pattern was observed for *Cpt1a* (CPT‐I) mRNA levels, thus corroborating the limited fatty acid oxidation in C57Bl/6J mice that were fed the HFD.

Interestingly, six of the 24 genes that were evaluated by RT‐qPCR were differentially expressed in the liver in both C57Bl/6J and FVB/N mice that were fed the chow diet. Genes that promote TAG re‐esterification (*Dgat‐2*) and fatty acid oxidation (*Ppara* and *Acox2*) were upregulated, suggesting fatty acid cycling within hepatocytes, with increases in synthesis and degradation. The lower expression of *Apob* (apoB) supports this possibility, in which lipids accumulate within hepatocytes that have this profile (Table 3), although the histological analysis did not reveal lipid accumulation in the liver in chow‐fed FVB/N mice.

With regard to glucose metabolism, *Cebpa* (C/EBP*α*) was upregulated 1.4‐times (Table 3). C/EBP*α* has been suggested to play a direct repressor role in *Scd‐1* promoter activity (Xu et al. 2016). Insulin receptor expression was downregulated 1.4‐times, suggesting lower insulin sensitivity in the liver in chow‐fed FVB/N mice (Table 3).

Despite the higher cholesterol levels that were observed in chow‐fed FVB/N mice compared with chow‐fed C57Bl/6J mice (Table 1), no differences in the cholesterol metabolism gene expression profile were found (Table 3).

Only four of the 24 genes that were analyzed were modulated by the HFD in FVB/N mice (*Scd1*,* Acox2*,* Insig2*, and *Irs2*). The expression of these genes *per se* cannot entirely explain the higher susceptibility to steatosis in HFD‐fed FVB/N mice. High *Scd1* expression is considered to promote lipid accumulation, and *Scd1* has been considered a target for the treatment of liver steatosis (Narce et al. 2012). In our model, *Scd1* expression decreased ~12‐times (Table 3). Importantly, gene regulation is only a starting point to understand the overall cellular response. Further western blot analyses and studies of enzymatic activity are necessary to better comprehend these findings.

## Discussion

Previous studies compared the C57Bl/6J and FVB/N mouse lines and the relevance of their genetic background to atherosclerosis in a model of *apoE* deficiency (Dansky et al. 1999), adipose tissue hypoxia, angiogenesis, and inflammation (Kim et al. 2013). In these studies, the animals received a regular chow diet or a high‐fat, high‐cholesterol diet. Dansky et al. (1999) reported higher susceptibility to atherosclerosis in apoE‐deficient C57Bl/6J mice and higher levels of proatherogenic factors in *apoE*‐deficient FVB/N mice (Dansky et al. 1999). Thus, the genetic background was clearly responsible for the observed differences in atherosclerosis susceptibility. Kim et al. (2013) reported greater remodeling capacity in WAT in C57Bl/6J mice compared with FVB/N mice (Kim et al. 2013).

Other authors have reported strain‐specific glucose metabolism under chow diet conditions (Berglund et al. 2008), strain‐specific insulin secretory function in response to a HFD (Andrikopoulos et al. 2005), and strain‐specific insulin resistance in DIO (Montgomery et al. 2013).

In this study, we found significant differences between the two mouse strains that were fed the chow diet and HFD. We confirmed DIO in HFD‐fed C57Bl/6J mice and lower fat accumulation in visceral fat depots in HFD‐fed FVB/N mice but no changes in subcutaneous fat depots. Furthermore, adipocytes were smaller in C57Bl/6J mice compared with FVB/N mice that were fed the chow diet. These findings are consistent with a previous study (Kim et al. 2013). However, in contrast to Kim et al. (2013), we observed nearly 30% weight gain in response to the HFD in FVB/N mice compared to chow counterpart. A similar effect of background was observed in a model of *apoE* deficiency (Dansky et al. 1999), in which FVB/N mice fed a chow diet had higher cholesterol and TAGs compared with C57Bl/6J mice that were also fed a chow diet. Interestingly, we found that FVB/N mice were heavier than C57Bl/6J mice and had larger fat depots when fed the chow diet (Table 1). These findings corroborate Berglund et al. (2008). In this study, however, FVB/N mice exhibited much higher HOMA‐IR, whereas QUICKI was similar. Furthermore, in Berglund et al. (2008), glucose disposal capacity in FVB/N mice was lower than in the other mouse strains, including C57BL/6J, thus corroborating our findings in the GTT (Fig. 1).

To gain further insights into whether FVB/N is a DIO‐prone or ‐resistant strain, further analyses revealed profound deleterious effects of the HFD in this lineage, and these effects were even more pronounced than in the C57Bl/6J strain. Glucose intolerance in FVB/N mice was worsened by the HFD. Notably, we used only half of the usual dose of glucose that is employed in the GTT because the full dose (2 g/kg) would have induced blood glucose levels that are above the detection limit (600 mg/dL) of our monitor.

Unexpected severe inflammation was found in epiWAT in FVB/N mice that were fed the HFD. Gene markers further corroborated the high degree of inflammation. We did not anticipate such a finding because tissue weight was not different between chow‐ and HFD‐fed FVB/N mice. RT‐PCR revealed increases in the mRNA levels of *Ccl2* (MCP‐1), *Lep* (leptin), and *Il6* (IL6) in WAT in chow‐fed FBV/N mice compared with chow‐fed C57Bl/6J mice. These findings contradict previous results (Kim et al. 2013), in which C57Bl/6J mice that were fed a chow diet exhibited greater inflammation in adipose tissue compared with chow‐fed FVB/N mice. Such a difference could be age‐related. We used 12‐week‐old animals, and Kim et al. (2013) used 9‐week‐old animals. Montgomery et al. (Montgomery et al. 2013) compared the responses of different mouse strains to a HFD. Our data, however, differ from this previous study in some aspects, such as similar weight and fat mass between chow‐fed C57Bl/6J and FVB/N mice. However, higher plasma TAG levels were found in FVB/N mice in both studies (Table 1). Both this study and Montgomery study showed higher body weight, fasting insulin, and glucose in HFD‐fed C57Bl/6J and FVB/N mice (Table 1). A similar pattern was observed with regard to the TAG response. C57Bl/6J mice did not exhibit changes in TAG levels in response to the HFD, whereas lower plasma/serum TAG levels were observed in HFD‐fed FVB/N mice (Table 1). Importantly, Montgomery et al. (2013) found that epiWAT was altered by the HFD in FVB/N mice, whereas no changes in liver weight were observed. In this study, the weight of epiWAT was similar in both chow‐ and HFD‐fed FVB/N mice, and the liver was larger in HFD‐fed FVB/N mice (Table 1). Additionally, subWAT presented HFD‐induced alterations in FVB/N mice, reflected by a greater weight (Table 1) and histological changes (not shown) that indicated a depot‐specific response. We observed a different response in FVB/N mice, in which an epiWAT inflammation profile and severe liver steatosis were evident.

Such distinct phenotypes between C57Bl/6J and FVB/N mice may be related to metabolism. Therefore, we decided to investigate genes that are related to metabolism in WAT and the liver. The main difference in WAT between these two strains that were both fed the chow diet was the upregulation of genes that are related to fatty acid synthesis (i.e., *Acaca* [ACC*α*] and *Fasn* [Fas]) and degradation (*Cpt1a* [CPT‐I] and *Ucp2*; Table 2). This upregulation is compatible with increases in lipogenesis in WAT and lipolysis (Coleman and Lee 2004; Strable and Ntambi 2010). Such cycling would lead to higher TAGs and free fatty acids in blood (Millward et al. 2010). If so, then the liver may be recruited to manage cholesterol and TAG overload. Further investigations of metabolism‐related genes in the liver revealed nearly no differences between C57Bl/6J and FVB/N mice. Notable changes, however, included the 1.7‐times upregulation of *Dgat‐2* (which suggests an increase in TAG re‐esterification; (Zammit 2013), 1.4‐times upregulation of *Ppara* (PPAR*α*; a key player in fatty acid oxidation in the liver; (Nakamura et al. 2014), and 1.6‐times downregulation of *Apob* (apoB). This pattern of basal gene expression suggests that the FVB/N mouse line is prone to lipid steatosis.

Insulin signaling plays a key role in determining intermediate metabolism (Saltiel and Kahn 2001). Insulin resistance alone may be responsible for the different energy flux between the FVB/N and C57Bl/6J strains. It is well known that even in insulin‐resistant animals, carbohydrates alone can stimulate insulin‐dependent metabolism via carbohydrate response element‐binding proteins ((Ishii et al. 2004). The FVB/N lineage is well known to present insulin resistance in the liver. We speculate that insulin resistance in the liver evokes prolipogenic energy flux, resulting in fat accumulation in FVB/N mice that are fed a chow diet.

When fed the HFD, C57Bl/6J mice presented a shift in energy flux. As they became more insulin‐resistant, some similarities to chow‐fed FVB/N mice were observed, including increases in adiposity and blood cholesterol. FVB/N mice that were fed the HFD exhibited a fatty liver, severe inflammation in epiWAT, and increases in the expression of proinflammatory cytokine genes, such as *Tnf* (TNF*α*) and *Il6* (IL6).

Altogether, our data challenge the notion that the FVB/N mouse strain is resistant to DIO. Such a purported DIO‐resistant status of FVB/N mice may mislead researchers who instead focus on the C57Bl/6J strain as the gold standard in studies of obesity. This situation is complicated further when transgenic models are generated by backcrossing FVB/N mice onto a C57Bl/6J background.

In summary, the FVB/N mouse line can help elucidate certain aspects of obesity and metabolic disorders beyond those that are established in the C57Bl/6J strain. The complete sequencing of the FVB/NJ mouse genome (Wong et al. 2012) will help reveal the genetic mechanisms that underlie different metabolic phenotypes.

## Conflict of interest

The authors declare no competing financial interests in relation to the work described.
